# Animal naming test stratifies the risk of falls and fall-related fractures in patients with cirrhosis

**DOI:** 10.1038/s41598-024-54951-8

**Published:** 2024-02-21

**Authors:** Takao Miwa, Tatsunori Hanai, Sachiyo Hirata, Kayoko Nishimura, Shinji Unome, Yuki Nakahata, Kenji Imai, Yohei Shirakami, Atsushi Suetsugu, Koji Takai, Masahito Shimizu

**Affiliations:** 1 Department of Gastroenterology/Internal Medicine, Graduate School of Medicine, Gifu University, 1-1 Yanagido, Gifu, 501-1194 Japan; 2 Center for Nutrition Support and Infection Control, Gifu University Hospital, Gifu, Japan; 3 Department of Gastroenterology, Asahi University Hospital, Gifu, Japan; 4 Division for Regional Cancer Control, Graduate School of Medicine, Gifu University, Gifu, Japan

**Keywords:** Gastroenterology, Medical research, Risk factors

## Abstract

This study aimed to determine the relationship between animal naming test (ANT), falls, and fall-related fractures in patients with cirrhosis. Cognitive impairment and frailty were assessed using ANT and Karnofsky performance status (KPS), respectively. Factors stratifying the risk of previous falls and fall-related fractures within 1 year were assessed using a logistic regression model. Factors affecting patient performance in ANT were evaluated using multiple regression analysis. Of the 94 patients, 19% and 5% experienced falls and fall-related fractures, respectively. The performance in ANT was worse in patients who experienced falls (11 vs. 18; *p* < 0.001) and fall-related fractures (8 vs. 16; *p* < 0.001) than in those who did not. After adjustment, females, KPS, and ANT (odds ratio [OR], 0.78; 95% confidence interval [CI], 0.65–0.93; *p* = 0.005) were associated with falls, while ANT was significantly associated with fall-related fractures (OR, 0.56; 95% CI 0.35–0.88; *p* = 0.012). Age and education affected the performance in ANT, whereas the use of Oriental zodiac did not. The ANT is useful for stratifying the risk of falls and fall-related fractures in patients with cirrhosis. The effects of age and education should be considered when applying ANT in the Japanese population.

## Introduction

Falls and fall-related injuries are a common healthcare problem worldwide, with approximately 30% of the older adults aged ≥ 60 years falling each year^1^. The main outcomes of falls are injuries, fractures, loss of mobility, and increased medical costs, which are major concerns^1^. A recent study reported that in 2017, more than 600,000 individuals died from falls worldwide^2^. Therefore, the guidelines on falls recommend screening individuals at high risk for falls with questionnaires and gait and balance tests, to provide a multifactorial approach to preventing falls and fall-related injuries^1^.

Because the ability to quickly react to external events is impaired in patients with cirrhosis, it is a major predisposing disease for falls and fall-related injuries, which can contribute to devastating outcomes^3,4^. Falls worsen the physical and mental condition and health-related quality of life of patients with cirrhosis^5,6^. Notably, recent studies have shown that falls increase the risk of severe injuries in patients with cirrhosis, leading to decreased independence and increased mortality^7,8^. Common risk factors for falls in patients include advanced age, previous history of falls, neuromuscular diseases, sedative medication use, and lower extremity weakness^4^. In addition, complications of cirrhosis such as sarcopenia, frailty, and hepatic encephalopathy (HE) can result in falls^4^.

Minimal/covert HE (MHE/CHE), the mildest form of HE, is a crucial risk factor for falls in patients with cirrhosis^9,10^. MHE/CHE is also associated with complications such as sarcopenia, occurrence of overt HE (OHE), reduced quality of life, and poor prognosis^11–14^. Given the clinical impact of MHE/CHE on these outcomes, screening is recommended for all patients with cirrhosis without previous OHE^12^. The gold-standard tests to investigate MHE/CHE are neuropsychological and neurophysiological tests, including the psychometric HE score (PHES) and computer-aided neuropsychiatric test^13–15^. The EncephalApp Stroop test has also been shown to be a useful tool to identify MHE/CHE and predict OHE^16–18^. Although these tests may be able to screen for MHE/CHE with a high risk of falls, the clinical application of these tests is limited because it is time consuming, requires a well-trained provider, and has limited availability.

Recently, the animal naming test (ANT) has been established as a point-of-care test to identify MHE/CHE in patients with cirrhosis^19^. The ANT is a simple neurological function test that asks patients to name as many animals as possible in one minute^19^. Since the test does not need any equipment and requires a short time of 60 s, the use of ANT is strongly recommended by the guidelines for HE^12^. However, data on the implementation of ANT in the Japanese population are limited, and in particular, the association between ANT and falls and fall-related fractures has not been examined.

The primary aim of this study was to investigate the association between ANT and falls and fall-related fractures in patients with cirrhosis. The secondary aim was to identify the factors affecting ANT performance in a Japanese cohort.

## Methods

### Study design and patients

This cross-sectional study involved 94 patients with cirrhosis who were admitted to Gifu University Hospital (Gifu, Japan) between December 2021 and June 2023. These patients were enrolled in a prospective observational study investigating ANT. The study protocol was reviewed and approved by the Institutional Review Board of the Graduate School of Medicine, Gifu University (approval number: 2021-B150), and was conducted in accordance with the Declaration of Helsinki. Written informed consent was obtained from all participants after the study objectives were explained by the health care professionals.

The study included patients aged ≥ 20 years with cirrhosis of any etiology. The exclusion criteria were patients with previous transjugular intrahepatic portosystemic shunt procedure, a history liver or any other organ transplantation, active infection within 1 month, OHE occurrence within 1 month, gastrointestinal bleeding within 1 month, alcohol abuse (≥ 60 g) within 1 month, neurological diseases, dementia, and life-threatening comorbidities, such as heart, respiratory, and renal failure, all of which may affect ANT performance.

### Outcomes and variables

The primary outcome was identification of factors associated with previous falls and fall-related fractures in patients with cirrhosis. Information on the history of falls and fall-related fractures from 1 year prior to admission until the time of hospitalization was collected through an interview on admission. The secondary aim was to clarify factors affecting ANT performance in a Japanese population.

Cirrhosis can be attributed to histology, biochemical parameters, imaging, and clinical symptoms. Clinical variables, including Child–Pugh score, model for end-stage liver disease (MELD) score, and biochemical parameters, were assessed on admission. HE was graded based on the West Haven criteria^13^.

### Assessment of ANT

ANT was evaluated within 1 week of admission according to a previously published method^19^. The patients were asked to list as many animals as they could within one minute. The results of ANT (number of animal names that could be listed) were calculated by excluding all repetitions and errors. Considering the effect of age and education on test performance^19^, a questionnaire was administered regarding the number of years of education following compulsory education, and the results were evaluated for junior high school graduates, high school graduates, and university graduates.

### Assessment of Karnofsky performance status

The Karnofsky performance status (KPS) is a validated tool to assess functional impairment and frailty in patients with cirrhosis^20^. Using the KPS, the patients were assigned to one of 10 categories ranging from 0 (death) to 100 (normal activity) based on the guidelines for frailty^20^.

### Assessment of Oriental zodiac

The Oriental zodiac is a cycle of 12 animals that represent different years, which is used in Japan to mark years in the same way as the Gregorian calendar. The 12 animals are rat, ox, tiger, rabbit, dragon, snake, horse, sheep, monkey, rooster, dog, and boar. Since the use of Oriental zodiac may affect the performance in ANT, patients were asked whether they recalled Oriental zodiac when answering the test.

### Statistical methods

Quantitative variables are expressed as medians (interquartile ranges) and qualitative variables are expressed as numbers (percentages). The differences between groups were compared using Mann–Whitney U test, Kruskal–Wallis test, or chi-squared test. Factors associated with falls and fall-related fractures were assessed using logistic regression analysis and the results are expressed as odds ratios (ORs) with 95% confidence intervals (CIs). The discriminative ability was assessed using receiver operating curve (ROC) analysis, and the results are expressed as area under the curve (AUC) with 95% CIs. Youden’s index was applied to evaluate the optimal cutoff values to identify the outcomes, and sensitivity and specificity were expressed based on the cutoff values. Factors affecting ANT performance in the Japanese cohort were assessed using multiple regression analysis. The variables used in the analyses were selected based on their clinical relevance and confounders.

In all analyses, the *p*-values were two-tailed and statistical significance was set at *p* < 0.05. Statistical analyses were performed using JMP Pro (version 17.0.0; SAS Institute Inc., Cary, NC, USA) and R (version 4.1.3; R Foundation for Statistical Computing, Vienna, Austria) software.

## Results

### Clinical characteristics of patients with cirrhosis

The clinical characteristics of patients with cirrhosis, included in this study, are shown in Table 1. The median age of the 94 patients included in the analysis was 72 years, and 30% were female. In terms of education, 29 (31%) were junior high school graduates, 48 (51%) were high school graduates, and 17 (18%) were university graduates. Among the participants, 58 (61%) had hepatocellular carcinoma and three (3%) had previous OHE. The median Child–Pugh and MELD scores were 5 and 7, respectively. The median ANT score was 16 points, and eight (9%) patients recalled the Oriental zodiac during the test.

**Table 1:** Baseline characteristics of patients with cirrhosis based on falls.

Characteristic	All patients (n = 94)	No falls (n = 76)	Falls (n = 18)	*p-*value	No fractures (n = 89)	Fractures (n = 5)	*p-*value
Age (years)	72 (65–78)	70 (63–76)	79 (73–81)	< 0.001	71 (65–78)	79 (73–83)	0.034
Female, n (%)	28 (30)	18 (24)	10 (56)	0.008	25 (28)	3 (60)	0.129
Body mass index (kg/m^2^)	24.0 (22.3–26.2)	24.5 (22.5–26.3)	23.4 (21.0–24.7)	0.180	24.0 (22.4–26.3)	23.3 (20.4–24.0)	0.235
Education, n (%)				0.041			0.281
Junior high school graduate	29 (31)	19 (25)	10 (56)		26 (29)	3 (60)	
High school graduate	48 (51)	42 (55)	6 (33)		46 (52)	2 (40)	
University graduate	17 (18)	15 (20)	2 (11)		17 (19)	0 (0)	
Etiology (HBV/HCV/ALD/Others), n	12/17/26/38	11/12/23/30	2/5/3/8	0.505	12/17/26/34	1/0/0/4	0.203
Hepatocellular carcinoma, n (%)	58 (62)	46 (61)	12 (67)	0.630	54 (61)	4 (80)	0.387
Previous OHE	3 (3)	2 (3)	1 (6)	0.526	2 (2)	1 (20)	0.028
Child–Pugh score	5 (5–7)	5 (5–7)	6 (5–6)	0.691	5 (5–7)	6 (5–7)	0.717
Child–Pugh class (A/B/C), n	70/19/5	56/17/3	2014/2/2	0.309	66/18/5	4/1/0	0.860
Laboratory test							
MELD score	7 (6–9)	7 (6–9)	7 (6–10)	0.684	7 (6–9)	7 (7–10)	0.750
International normalized ratio	1.01 (0.95–1.09)	1.01 (0.95–1.10)	1.00 (0.91–1.03)	0.495	1.00 (0.94–1.09)	1.02 (0.96–1.10)	0.643
Platelet (10^9^/L)	147 (93–181)	150 (92–180)	143 (105–183)	0.799	150 (95–182)	91 (165–168)	0.192
Creatinine (mg/dL)	0.80 (0.65–0.91)	0.80 (0.66–0.96)	0.75 (0.60–0.85)	0.235	0.80 (0.66–0.92)	0.74 (0.52–0.96)	0.409
Albumin (g/dL)	3.9 (3.2–4.2)	3.9 (3.2–4.3)	3.7 (3.1–4.0)	0.127	3.9 (3.3–4.2)	3.1 (3.0–4.0)	0.169
Bilirubin (mg/dL)	0.9 (0.7–1.4)	0.9 (0.7–1.4)	1.0 (0.7–1.5)	0.512	0.9 (0.7–1.4)	1.2 (0.9–1.9)	0.233
Sodium (meq/L)	139 (137–140)	139 (137–140)	140 (137–141)	0.954	139 (137–140)	136 (132–140)	0.084
Ammonia (μg/dL)	61 (45–86)	63 (44–85)	58 (44–93)	0.737	60 (44–84)	81 (50–128)	0.257
Karnofsky performance status	100 (90–100)	100 (90–100)	90 (90–100)	0.010	100 (90–100)	90 (90–100)	0.373
Animal naming test	16 (13–20)	18 (13–21)	11 (8–15)	< 0.001	16 (13–21)	8 (6–11)	< 0.001
Oriental zodiac use, n (%)	8 (9)	6 (8)	2 (11)	0.660	7 (8)	1 (20)	0.344

Values are presented as number (percentage) or median (interquartile range). Statistical analyses were performed using the chi-square test or Mann–Whitney U test.

ALD, alcohol-related liver disease; HBV, hepatitis B virus; HCV, hepatitis C virus; MELD, model for end-stage liver disease; OHE, overt hepatic encephalopathy.

Of the patients analyzed, 18 (19%) and five (5%) had experienced falls and fall-related fractures (fracture sites: two lower limbs, one upper limb, one vertebral body, and one rib), respectively, from 1 year prior to admission until the time of hospitalization. Patients who experienced falls had significantly poorer performance in ANT than those who did not (median, 11 vs. 18; *p* < 0.001). In addition, patients with falls were characterized by older age, a higher proportion of females, a lower educational background, and lower KPS scores than those without falls. Similarly, patients with fall-related fractures were older, had a previous OHE, and had worse performance in ANT (median, 8 vs. 16; *p* < 0.001) than those without fractures (Table 1).

### Factors associated with falls and fall-related fractures

In the univariate analysis, age, female sex, education, KPS, and ANT were significantly associated with falls (Supplementary Table S1). Multivariate analysis showed that females (OR, 5.15; 95% CI 1.33–19.94; *p* = 0.018), KPS (OR, 0.91; 95% CI 0.83–0.99; *p* = 0.029), and ANT (OR, 0.78; 95% CI 0.65–0.93; *p* = 0.005) were independently associated with falls in patients with cirrhosis (Table 2). For fall-related fractures, univariate analysis revealed that ANT was the only significant factor in patients with cirrhosis (Supplementary Table S2). Multivariate analysis confirmed that the association between ANT and fall-related fractures remained significant even after adjusting for age (OR, 0.56; 95% CI 0.35–0.88; *p* = 0.012) (Table 2).

**Table 2:** Multivariate analysis of factors associated with falls and fall-related fractures in patients with cirrhosis.

Characteristic	OR (95% CI)	*p-*value
Fall		
Age (years)	1.04 (0.96–1.13)	0.293
Female	5.15 (1.33–19.94)	0.018
Karnofsky performance status	0.91 (0.83–0.99)	0.029
Animal naming test	0.78 (0.65–0.93)	0.005
Fall-related fracture		
Age (years)	0.97 (0.95–1.11)	0.682
Animal naming test	0.56 (0.35–0.88)	0.012

Multivariate analysis was performed using the logistic regression model.

CI, confidence interval; OR, odds ratio.

### Ability of ANT to identify falls and fall-related fractures

**Figure 1:**
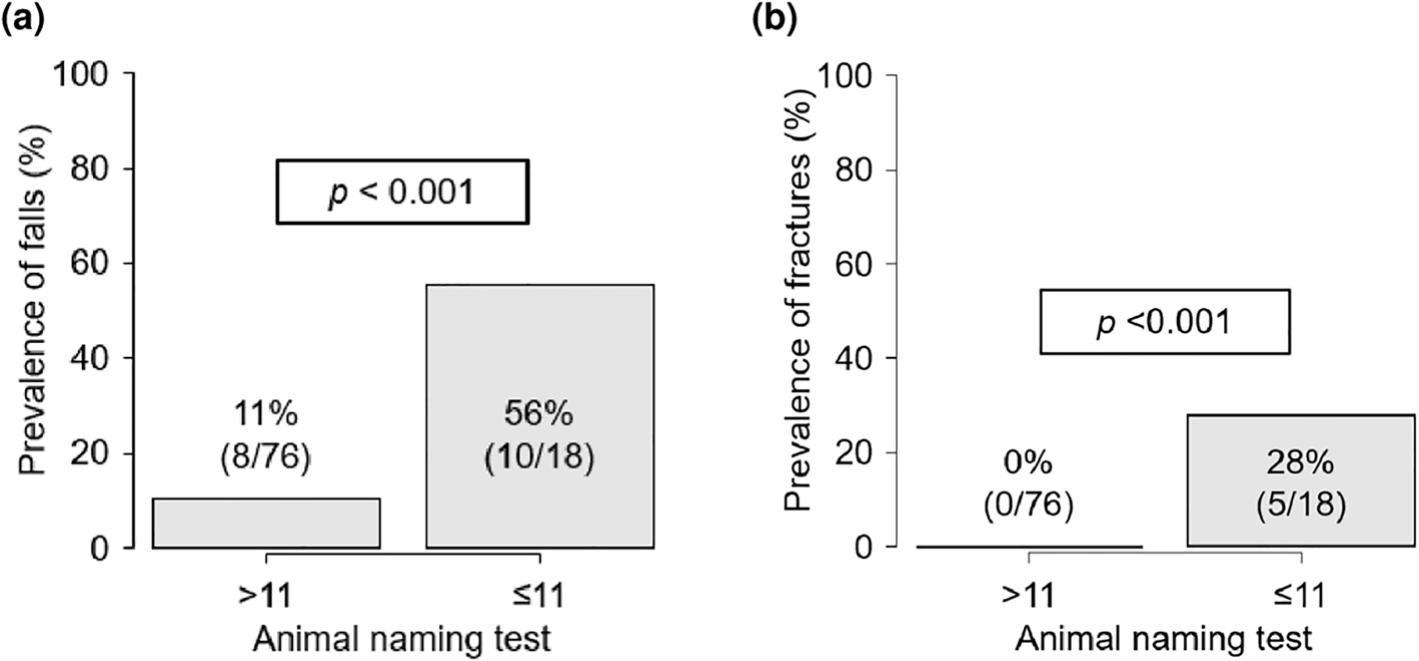
The association between ANT, falls, and fall-related fractures in patients with cirrhosis. The prevalence of (**a**) falls and (**b**) fall-related fractures were significantly higher in patients with ANT scores ≤ 11 than in those with ANT scores > 11. ANT, animal naming test.

The ROC analysis showed that ANT had a good ability to identify falls (AUC, 0.82; 95% CI 0.72–0.92) and fall-related fractures (AUC, 0.95; 95% CI 0.90–1.00), with optimal cutoff values for both outcomes of 11. Based on the cutoff value of ANT ≤ 11, the sensitivity and specificity were 0.89 and 0.56, respectively, to identify falls, and 0.85 and 1.00, respectively, for fall-related fractures. Patients with ANT ≤ 11 had a significantly higher prevalence of falls (56 vs. 11%; *p* < 0.001; Fig. 1a) and fall-related fractures (28 vs. 0%; *p* < 0.001; Fig. 1b) than those with ANT > 11. In addition, females had a higher prevalence of falls than males (36 vs. 12%; *p* = 0.008).

Considering the frailty scale, the ROC analysis of KPS (AUC, 0.67; 95% CI 0.53–0.80) showed that KPS ≤ 90 was the optimal cutoff value to identify falls in patients with cirrhosis (sensitivity, 0.61 and specificity, 0.71). Patients with KPS ≤ 90 had a higher prevalence of falls than those with KPS = 100 (33 vs. 11%; *p* = 0.010).

### The performance of Japanese patients with cirrhosis in ANT

**Figure 2:**
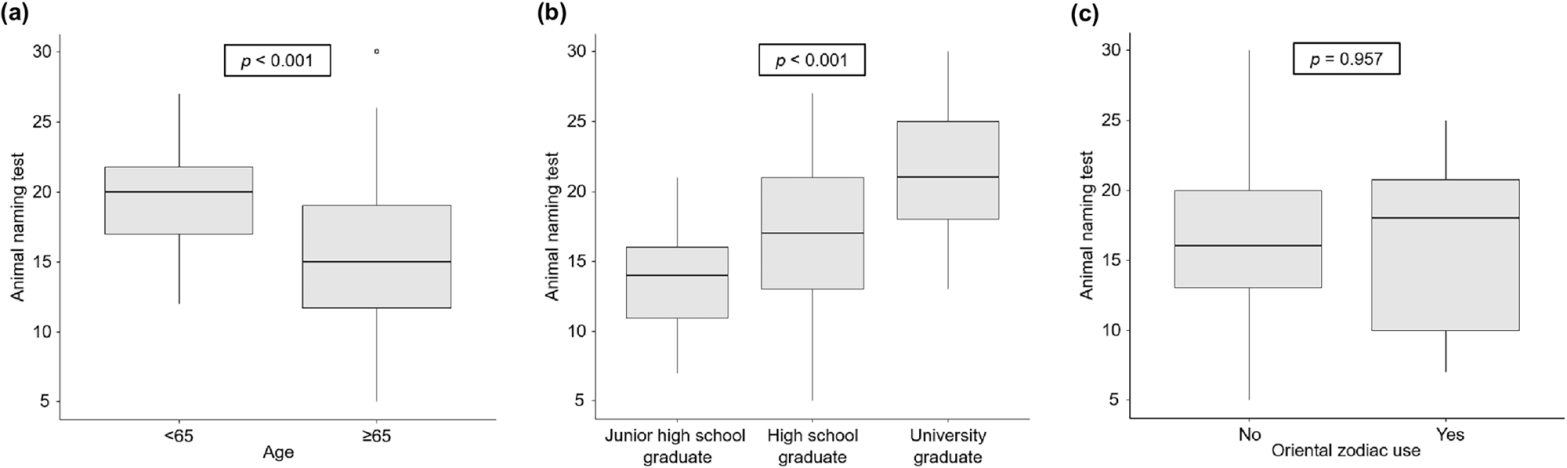
Factors affecting the performance of Japanese patients with cirrhosis in ANT. The performance in ANT was compared according to groups divided by (**a**) age, (**b**) education, and (**c**) Oriental zodiac use. ANT, animal naming test.

Multiple regression analysis was performed to reveal the factors associated with the performance of Japanese patients with cirrhosis in ANT (Table 3). The results showed that age (*β*, -0.013; *p* = 0.002) and education years (*β*, 0.433; *p* = 0.007) independently influenced the performance in ANT, whereas Oriental zodiac did not (*β*, − 0.011; *p* = 0.849). Patients aged ≥ 65 had significantly lower ANT scores than those aged < 65 years (median, 15 vs. 20; *p* < 0.001; Fig. 2a). Furthermore, the ANT scores significantly improved as the education increased, from junior high school to high school to university graduates (median, 14 vs. 17 vs. 21; *p* < 0.001; Fig. 2b). In contrast, the use of Oriental zodiac did not affect ANT scores in Japanese patients with cirrhosis (median, 16 vs. 18; *p* = 0.957; Fig. 2c).

**Table 3:** Multiple regression analysis of factors associated with performance in animal naming test.

Characteristic	*Β*	SE* (B)*	*β*	*t*	*p-*value
Age (years)	− 0.153	0.049	− 0.013	− 3.147	0.002
Sex	− 0.460	1.106	− 1.027	− 0.416	0.678
Body mass index (kg/m^2^)	− 0.012	0.129	− 0.018	− 0.092	0.927
Education (years)	0.580	0.210	0.433	2.766	0.007
Previous OHE	− 1.833	2.996	− 0.623	− 0.612	0.542
Child–Pugh score	0.210	0.346	0.011	0.605	0.547
Karnofsky performance status	0.082	0.074	0.039	1.104	0.273
Oriental zodiac use	− 0.342	1.789	− 0.011	− 0.191	0.849

Values are presented as unstandardized beta (*Β*), the SE for *B*, the standardized beta (*β*), and the t-test statistic (*t*). Statistical analysis was performed using multiple regression analysis (*R*^2^ = 0.302; adjusted *R*^2^ = 0.236; *F* [8, 85] = 4.589); *p* < 0.001).

OHE, overt hepatic encephalopathy; SE, standard error.

## Discussion

Screening high-risk groups for falls is critical because trauma and fractures from falls have a significant impact on the health-related quality of life and prognosis of patients with cirrhosis. The results of this study revealed that approximately 19% of patients with cirrhosis experienced falls within 1 year, which appears comparable to those of a previous study that showed that 22% of patients with cirrhosis had fallen within 1 year^9^. Prospective studies have also confirmed that the prevalence of future falls within 1 year in patients with cirrhosis is 18–29%^8,10^, which is consistent with our results. Furthermore, given that patients with cirrhosis are currently older^21^, the burden of falls is expected to increase further in these patients. Therefore, there is growing clinical demand for a simple screening tool that can identify patients with cirrhosis who are at a high risk of falls.

The primary finding of the present study is that ANT is a valuable tool for assessing the risk of falls and fall-related fractures in patients with cirrhosis. Generally, cognitive impairment increases the risk of falls in the elderly^22^. Particularly in patients with cirrhosis, even mild cognitive impairment such as MHE/CHE is strongly associated with falls^9,10^. MHE/CHE is involved in falls independent of a history of OHE and antidepressant therapy^9^. MHE/CHE is also the only independent factor that can predict future falls in patients with cirrhosis^10^. PHES was used to diagnose MHE/CHE in these reports^9,10^, indicating the usefulness of this gold-standard method in examining the risk of falls in patients with cirrhosis. However, this is the first study to show that ANT, a simple point-of-care test, was useful in assessing the risk of falls and fall-related fractures in patients with cirrhosis. Indeed, ANT showed good ability to stratify the risk of falls (AUC, 0.82) and fall-related fractures (AUC, 0.95). Interestingly, ANT has been reported to be a useful assessment tool for patients with cirrhosis, reflecting not only MHE/CHE but also frailty and patient-reported outcomes^23^. In fact, there was a significant correlation between ANT and handgrip strength in patients with cirrhosis included in our study (*r* = 0.33; *p* = 0.001 [n = 93]). In addition, the cutoff value of ANT ≤ 11 for stratifying the risk of falls and fall-related fractures in the present study was lower than the previously reported cutoff values for MHE/CHE (ANT score of 15–20), but similar to those for frailty and patient-reported outcomes (ANT < 10)^19,24,25^. Therefore, ANT may perceive fall risk by assessing not just cognitive impairment but also frailty.

The present study also revealed that female sex and a lower frailty score in KPS were associated with a higher risk of falls in patients with cirrhosis. Female sex and frailty are well known risk factors for falls in older adults^26^. The present results are consistent with those of previous studies reported that female patients are at a higher risk of falls than males patients with cirrhosis^9,10^. Frailty leads to health disadvantages in patients with cirrhosis, including reduced quality of life, hepatic decompensation, and increased mortality^20^. KPS is the gold-standard method to assess frailty, recommended by the guidelines for cirrhosis^20^. Our study included patients with relatively preserved mobility and none of them were using mobility aids. Therefore, the results suggest that even mild mobility decline can increase the risk of falls in patients with cirrhosis. The results are supported by previous studies, which evaluated frailty scales such as handgrip strength and chair-stand and their impact on fall risks in patients with cirrhosis^8,27^. Our study has important implications for risk assessment of falls in patients with cirrhosis, and patients at high risk of falls require detailed assessment of frailty and multifactorial intervention to prevent critical injuries and enhance outcomes^1^.

The secondary important finding of this study was that the performance of Japanese patients with cirrhosis in ANT is influenced by age and education, but not by zodiac use. The results of our study are consistent with those of previous studies. Aging is a robust factor that affects the results of MHE/CHE testing^15,18^. In addition, a previous Italian study showed that older age and education affected the performance in ANT and this study suggested simplified ANT adjusted for age and education^19^. The simplified ANT was defined to add 3 animals for patients with education < 8 years and aged ≤ 80 years and add 6 animals for those with education < 8 years and aged > 80 years to assess MHE/CHE in patients with cirrhosis^19^. Since our results showed that older age is associated with ANT performance, it is reasonable to adjust the results of ANT according to age in Japanese patients with cirrhosis. However, the adjustment according to education < 8 years is not applicable to Japanese patients with cirrhosis because the compulsory education period in Japan is 9 years, including elementary and junior high schools, with an almost 100% enrollment rate^28^. Furthermore, the rate of progression to high school exceeds 95%^28^. In fact, none of the participants in our study had < 8 years of education, which requires adjustment in simplified ANT^19^. Moreover, the Japanese are familiar with Oriental zodiac animals, and cultural differences can potentially influence the performance in ANT. Overall, the results of the present study suggest that the effects of age and education should be considered when assessing ANT in Japanese patients with cirrhosis. To date, no study has evaluated the association between ANT and MHE/CHE diagnosed with a gold-standard method in Japanese patients with cirrhosis. In a limited data set (n = 45), a significant correlation was found between the number connection test-A (*r* = -0.36; *p* = 0.015), number connection test-B (*r* = -0.46; *p* = 0.001), and ANT in our study. Therefore, further studies are required to establish the normative value of ANT for assessing MHE/CHE in these patients.

This study had the following limitations: First, this is a single-center cross-sectional study assessing previous falls and fall-related fractures, multicenter prospective studies are required to evaluate the impact of ANT on future outcomes in patients with cirrhosis. In addition, unmeasured confounding bias may affect the results of our study. Second, we evaluated only ANT and other tests for MHE/CHE were not performed. Third, the small number of participants and outcomes may have limited the statistical power of this study. Fourth, since our study did not include controls, the difference in the performance in ANT, falls, and fall-related fractures between those with and without cirrhosis in the Japanese population remains unclear. Therefore, we are planning further studies to evaluate the association between falls, fall-related fractures, and gold-standard diagnosis of MHE/CHE in Japanese patients with cirrhosis. Despite these limitations, the strength of the present study should be emphasized by the first evidence on the ANT in Japanese patients with cirrhosis and its relationship with falls and fall-related fractures.

In conclusion, ANT, a simple bedside test, is a useful cognitive assessment tool for stratifying the risk of falls and fall-related fractures in patients with cirrhosis. When applying ANT in Japanese patients with cirrhosis, the influence of age and education should be considered.

### Supplementary Information

Supplementary Tables.

## Data Availability

The data analyzed in this study is available from the corresponding author on reasonable request.
