# Supplementary material for: Animal naming test stratifies the risk of falls and fall-related fractures in patients with cirrhosis
Source: Sci Rep. 2024 Feb 21;14:4307. doi: 10.1038/s41598-024-54951-8 (PMC10881459; doi:10.1038/s41598-024-54951-8)
Supplement: Supplementary file 1 — Supplementary Tables. [file 41598_2024_54951_MOESM1_ESM.pdf]

Supplementary Information

Title: Animal naming test stratifies the risk of falls and fall-related fractures in patients with cirrhosis

Takao Miwa[1], Tatsunori Hanai[1,2], Sachiyo Hirata[2], Kayoko Nishimura[2], Shinji Unome[1], Yuki Nakahata[1,3], Kenji Imai[1], Yohei Shirakami[1], Atsushi Suetsugu[1], Koji Takai[1,4], Masahito Shimizu[1]

1. Department of Gastroenterology/Internal Medicine, Graduate School of Medicine, Gifu University, Gifu, Japan
2. Center for Nutrition Support and Infection Control, Gifu University Hospital, Gifu, Japan
3. Department of Gastroenterology, Asahi University Hospital, Gifu, Japan
4. Division for Regional Cancer Control, Graduate School of Medicine, Gifu University, Gifu, Japan

Supplementary Table S1. Univariate analysis of factors associated with falls in patients with cirrhosis

Characteristic	OR (95% CI)	*p*-value*
Age (years)	1.09 (1.02–1.16)	0.008
Female	4.03 (1.38–11.73)	0.011
Body mass index (kg/m^2)	0.91 (0.79–1.05)	0.184
Education, n (%)		
Junior high school graduate†	1.00	
High school graduate	0.27 (0.09–0.86)	0.026
University graduate	0.25 (0.05–1.34)	0.106
Etiology of cirrhosis		
HBV†	1.00	
HCV	2.29 (0.37–14.32)	0.376
ALD	0.72 (0.10–4.93)	0.736
Others	1.47 (0.27–8.00)	0.658
Hepatocellular carcinoma	1.30 (0.44–3.85)	0.631
Previous OHE	2.18 (0.19–25.42)	0.535
Child–Pugh score	1.11 (0.86–1.44)	0.411
MELD score	0.99 (0.84–1.16)	0.901
Karnofsky performance status	0.92 (0.87–0.98)	0.013
Animal naming test	0.76 (0.65–0.88)	<0.001
Oriental zodiac use	1.46 (0.27–7.90)	0.662

*Univariate analysis was performed using logistic regression model.

†Reference group.

Abbreviations: ALD, alcohol-related liver disease; CI, confidence interval; HBV, hepatitis B virus; HCV, hepatitis C virus; MELD, model for end-stage liver disease; OHE, overt hepatic encephalopathy; OR, odds ratio

Supplementary Table S2. Univariate analysis of factors associated with fall-related fractures in patients with cirrhosis

Characteristic	OR (95% CI)	*p*-value*
Age (years)	1.10 (0.99–1.23)	0.078
Female	3.84 (0.61–24.37)	0.154
Body mass index (kg/m^2)	0.86 (0.67–1.12)	0.268
Education, n (%)		
Junior high school graduate†	1.00	
High school graduate	0.38 (0.06–2.40)	0.302
University graduate	NA	NA
Etiology of cirrhosis		
HBV†	1.00	
HCV	NA	NA
ALD	NA	NA
Others	1.41 (0.14–13.91)	0.768
Hepatocellular carcinoma	2.59 (0.28–24.16)	0.403
Previous OHE	10.88 (0.81–146.61)	0.072
Child–Pugh score	0.96 (0.56–1.64)	0.871
MELD score	0.96 (0.871–1.31)	0.819
Karnofsky performance status	0.99 (0.90–1.08)	0.758
Animal naming test	0.58 (0.40–0.85)	0.005
Oriental zodiac use	2.93 (0.29–29.90)	0.365

*Univariate analysis was performed using the logistic regression model.

†Reference group.

Abbreviations: ALD, alcohol-related liver disease; CI, confidence interval; HBV, hepatitis B virus; HCV, hepatitis C virus; MELD, model for end-stage liver disease; NA, not available; OHE, overt hepatic encephalopathy; OR, odds ratio
